# Effects of Prone Positioning on Respiratory Mechanics and Oxygenation in Critically Ill Patients With COVID-19 Requiring Venovenous Extracorporeal Membrane Oxygenation

**DOI:** 10.3389/fmed.2021.810393

**Published:** 2022-01-17

**Authors:** Driss Laghlam, Julien Charpentier, Zakaria Ait Hamou, Lee S. Nguyen, Frédéric Pene, Alain Cariou, Jean-Paul Mira, Mathieu Jozwiak

**Affiliations:** ^1^Assistance Publique-Hôpitaux de Paris, Service de Médecine Intensive Réanimation, Hôpitaux Universitaires Paris-Centre, Hôpital Cochin, Paris, France; ^2^Université de Paris, Paris, France; ^3^Recherche et Innovation de la Clinique Ambroise Paré, Neuilly-Sur-Seine, France; ^4^Equipe 2 CARRES, UR2CA-Unité de Recherche Clinique Côte d'Azur, Université Côte d'Azur UCA, Nice, France

**Keywords:** acute respiratory distress syndrome (ARDS), alveolar recruitment, prone positioning (PP), venovenous extracorporeal membrane oxygenation, positive end-expiratory pressure (PEEP)

## Abstract

**Background:**

The effect of prone positioning (PP) on respiratory mechanics remains uncertain in patients with severe acute respiratory distress syndrome (ARDS) requiring venovenous extracorporeal membrane oxygenation (VV-ECMO).

**Methods:**

We prospectively analyzed the effects of PP on respiratory mechanics from continuous data with over a thousand time points during 16-h PP sessions in patients with COVID-19 and ARDS under VV-ECMO conditions. The evolution of respiratory mechanical and oxygenation parameters during the PP sessions was evaluated by dividing each PP session into four time quartiles: first quartile: 0–4 h, second quartile: 4–8 h, third quartile: 8–12 h, and fourth quartile: 12–16 h.

**Results:**

Overall, 38 PP sessions were performed in 10 patients, with 3 [2–5] PP sessions per patient. Seven (70%) patients were responders to at least one PP session. PP significantly increased the PaO_2_/FiO_2_ ratio by 14 ± 21% and compliance by 8 ± 15%, and significantly decreased the oxygenation index by 13 ± 18% and driving pressure by 8 ± 12%. The effects of PP on respiratory mechanics but not on oxygenation persisted after supine repositioning. PP-induced changes in different respiratory mechanical parameters and oxygenation started as early as the first-time quartile, without any difference in PP-induced changes among the different time quartiles. PP-induced changes in driving pressure (−14 ± 14 vs. −6 ± 10%, *p* = 0.04) and mechanical power (−11 ± 13 vs. −0.1 ± 12%, *p* = 0.02) were significantly higher in responders (increase in PaO_2_/FiO_2_ ratio > 20%) than in non-responder patients.

**Conclusions:**

In patients with COVID-19 and severe ARDS, PP under VV-ECMO conditions improved the respiratory mechanical and oxygenation parameters, and the effects of PP on respiratory mechanics persisted after supine repositioning.

## Background

Severe acute respiratory syndrome-coronavirus 2 (SARS-CoV2) is an emerging virus that has been responsible since December 2019 for the global coronavirus disease-2019 (COVID-19) pandemic. Most severe cases of respiratory involvement can lead to acute respiratory distress syndrome (ARDS), with high mortality of up to 60% (1–3).

The specific management of patients with moderate ARDS (ratio of arterial oxygen partial pressure (PaO_2_)/inspired oxygen fraction (FiO_2_) < 200) to severe (PaO_2_/FiO_2_ ratio < 100) consists, according to current recommendations, to consider prolonged (16 h) prone positioning (PP) session in patients with a P/F ratio < 150 despite optimal ventilatory management (4). The effects of PP are characterized by homogenization of transpulmonary pressure and distribution of total pulmonary stress and strain. These effects lead to decrease in alveolar overdistension of non-dependent pulmonary areas and reduction in cyclic opening-closing phenomena in dependent pulmonary areas (5). PP is also associated with improvement in ventilation-perfusion ratio, and participates in enhancement of oxygenation (5). In addition, PP is associated with lower patient mortality regardless of its effects on oxygenation (6).

In most severe patients, implantation of a venovenous extracorporeal membrane oxygenation (VV-ECMO) device can be proposed (7) and is currently integrated into the management algorithm of most severe ARDS (4). The use of VV-ECMO has shown a beneficial effect in patients with severe ARDS related to COVID-19 (8–12), as well as in patients with ARDS related to other viral diseases (Middle East Respiratory Syndrome coronavirus and H1N1 influenza) (13). The use of PP in patients with severe ARDS requiring VV-ECMO remains controversial (14). A retrospective analysis of the EOLIA study showed that using PP in patients under VV-ECMO allowed for higher rate of ECMO weaning and better survival rates (15), but another retrospective study contradicted these results by finding no effect of PP on these two parameters (16). So far, the effects of PP on respiratory mechanics in patients with severe ARDS requiring VV-ECMO remain uncertain and scarcely studied.

Thus, the main goal of this exploratory study was to assess the physiological respiratory effects of PP and evolution of respiratory mechanical and oxygenation parameters during PP sessions in patients with COVID-19 requiring VV-ECMO.

## Methods

### Patients

This prospective and descriptive single-center study was conducted in the 24-bed ICU of Cochin University Hospital. The study was approved by the Ethics Committee of the Société de Réanimation de Langue Française (CE SRLF 20-72). All the patients or next of kin were informed about the study and consented to participate.

We included all consecutive patients under mechanical ventilation with the following inclusion criteria: (i) presence of ARDS according to the Berlin definition (12); (ii) VV-ECMO implantation for patients with severe ARDS meeting one of the following criteria: (1) PaO_2_/FiO_2_ ratio less than 50 mmHg for longer than 3 h or (2) PaO_2_/FiO_2_ less than 80 mmHg for longer than 6 h, or (3) arterial pH < 7.25 with arterial carbon dioxide partial pressure (PaCO_2_) greater than or equal to 60 mmHg for longer than 6 h with respiratory rate of 35 breaths/min and mechanical ventilation settings adjusted to maintain plateau pressure (Pplat) less than or equal to 32 cm H_2_O (7); and (iii) positive COVID-19 real-time reverse transcriptase-polymerase chain reaction assay in nasal swabs or pulmonary samples. Exclusion criteria were the following: (i) patients < 18 years old, (ii) pregnancy, and (iii) patients under legal protection.

### ECMO Settings

All the patients were implanted using a percutaneous approach, either femoro-jugular or in femoro-femoral, with 19F jugular cannula and 23F femoral cannula. No blood gases were performed at the ECMO level. The ECMO flow was set to obtain a ECMO flow/cardiac output > 60% (17), and sweep gas flow was titrated to reach PaCO_2_ < 45 mmHg. The fraction of inspired oxygen in circuit was titrated to obtain arterial oxygenation ≥ 90 % (7). All the patients received curative anticoagulation with unfractionated heparin (with blood target anti-Xa between 0.30 and 0.5 UI/ml) (10). Patients were weaned off VV-ECMO when clinical and radiological improvement after a successful weaning test, as described in ECMO to rescue lung injury in severe ARDS (EOLIA) trial (7).

### Ventilatory Settings and Measurements

Before VV-ECMO implantation, all the patients were initially mechanically ventilated (CARESCAPE R860; GE Healthcare, Chicago, IL, United States, or EVITA V500 Infinity; DRAGER, Lübeck, Allemagne) in the volume assist-controlled mode or pressure regulated volume control mode. Tidal volume was set at 6 ml/kg of predicted body weight. Positive end-expiratory pressure (PEEP) level was titrated to reach a maximum Pplat of 30 cmH2O with a maximum driving pressure of 15 cmH2O. Respiratory rate and inspiratory/expiratory time ratio were adjusted to prevent hypercapnia and avoid dynamic intrinsic PEEP. FiO_2_ was titrated to obtain a peripheral oxygen saturation ≥ 90% (4). An airway humidification system was used in all the patients.

Under VV-ECMO, all the patients were ventilated in the volume assist-controlled mode. Ultraprotective ventilation was applied with the tidal volume set at 3 ml/kg of predicted body weight. The level of PEEP was titrated to reach a maximum Pplat of 25 cmH2O with a maximum driving pressure of 15 cmH2O (18). Respiratory rate and sweep gas were set to maintain PaCO_2_ < 45 mmHg. FiO_2_ and membrane oxygen fraction were titrated to obtain a peripheral oxygen saturation ≥ 90%. All the patients were sedated and received neuromuscular blockade agents.

Compliance of the respiratory system was calculated as tidal volume/(plateau pressure–total PEEP). Driving pressure was calculated as plateau pressure–total PEEP. Mechanical power was calculated as.098 × tidal volume × respiratory rate × peak pressure–driving pressure/2 (19, 20). Oxygenation index was calculated as (mean airway pressure × FiO_2_)/arterial partial oxygen pressure (PaO_2_).

### Prone Positioning Sessions

The decision to perform PP in patients under VV-ECMO was based on PaO_2_/FiO_2_ ratio value and left at the discretion of the attending physician, except in patients with persistent PaO_2_/FiO_2_ ratio < 100, on whom PP was systematically performed according to local practices. A minimum of five trained caregivers including one physician was required to perform PP, with two caregivers dedicated for maintaining cannulas. The PP sessions were performed as in the PROSEVA study: no thoraco-pelvic support was used, and the PP sessions were planned to last at least 16 h (6). During PP sessions, the VV-ECMO and ventilatory settings were unchanged. Patients with increase in PaO_2_/FiO_2_ ratio > 20% during PP sessions were considered as responders (21). According to local practices, enteral feeding was stopped 1 h before the onset of PP session, resumed at the previous rate during PP session, and then stopped again at the end of the PP session for 1 h after supine repositioning. In case of poor tolerance, enteral feeding was stopped during the whole PP session.

### Data Collection

Baseline characteristics, as well as clinical and biological data, were collected from the medical electronic sheets of the patients. VV-ECMO complications, therapeutics, vital status, and length of stay in intensive care unit (ICU) were also collected with a maximum follow-up to day 60.

Respiratory and hemodynamic parameters were continuously monitored with dedicated software, with a refreshing rate of two per minute (Clinisoft® GE Healthcare Centricity, Chicago, IL, United States). Thus, the analysis of the PP effects on respiratory mechanics and hemodynamics, and the analysis of the evolution of the respiratory mechanical parameters during PP sessions, were based on continuous data with over a thousand time points (22).

### Statistical Analysis

Since it was an exploratory study, no sample size calculation was performed *a priori*. The normality distribution of continuous variables was tested by Shapiro-Wilk test. Continuous variables were expressed as mean ± standard deviation or median [interquartile] according to data distribution and categorical variables as numbers (percentages). Comparisons between groups were performed by Mann-Whitney U or Student's *t*-tests for continuous variables and chi-2 or Fisher exact tests for categorical variables. Correlations were performed using Pearson or Spearman's correlation coefficients, according to data distribution.

The effects of PP on respiratory mechanics and their evolution during PP sessions were analyzed from continuously recorded respiratory data with over a thousand time points during PP sessions (22). The evolution of respiratory mechanical and oxygenation parameters during PP sessions was evaluated by dividing each PP session into four time quartiles as follows: first quartile: 0–4 h; second quartile: 4–8 h; third quartile: 8–12 h; and fourth quartile: 12–16 h. Comparisons of continuous respiratory variables before PP (i.e., during the 2 h before a PP session), during PP (i.e., during the 16 h of a PP session), and after supine repositioning (i.e. during the 2 h after supine repositioning) (23), as well as among the four quartiles of time of PP sessions were performed by two-way ANOVA for repeated measurements followed by paired Student *t*-tests with Bonferroni correction if needed, or by Friedman test followed by Wilcoxon tests if needed according to data distribution.

A *p* value < 0.05 was considered statistically significant. Statistical analysis was performed with MedCalc 11.6.0 (MedCalc, Mariakerke, East Flanders, Belgium).

## Results

### Patient Characteristics

Between March 1, 2020 and June 1, 2021, 261 patients with COVID-19 and ARDS were admitted in our ICU, and 24 (9%) required VV-ECMO: 18 (75%) of the patients were men, and two (8%) were immunocompromised (one patient with cystic fibrosis and one pregnant woman) (Table 1). All the patients received corticosteroids, and tocilizumab was administered in three (12%) patients.

**Table 1:** Characteristics and outcomes of patients requiring VV-ECMO.

	**Whole cohort** **(***n*** = 24)**	**Prone positioning** **(***n*** = 10)**	**No prone positioning** **(***n*** = 14)**	***p***
**Characteristics**				
Age (years)	52 [43–58]	52 [37–59]	52 [44–57]	0.74
Male (*n*,%)	18 (75)	7 (70)	11 (79)	0.67
Body mass index (kg/m^2^)	30 [25–34]	30 [29–35]	26[23–33]	0.13
Hypertension (*n*,%)	7 (29)	2 (20)	5 (36)	0.63
Diabetes mellitus (*n*,%)	7 (29)	1 (10)	6 (43)	0.08
Smoker (*n*,%)	4 (17)	1 (10)	3 (21)	0.61
Immunodepression (*n*,%)	2 (8)	1 (10)	1 (7)	0.80
SAPS–II	61 [29–75]	64 [49–72]	55 [27–77]	0.72
SOFA score	10 [6–14]	11 [6–14]	9 [5–15]	0.97
Onset of symptoms-intubation time (days)	7 [6–13]	7 [7–9]	8 [6–16]	0.21
Onset of symptoms-vv-ecmo time (days)	15 [12–22]	14 [11–19]	17 [14–25]	0.21
Intubation-vv-ecmo time (days)	6 [3–11]	5 [4–10]	6 [3–12]	0.92
**Ventilatory settings and management before vv-ecmo implantation**				
Tidal volume (ml/kg)	5.8 [5.2–6.2]	5.9 [5.1–6.4]	5.8 [4.9–6.1]	0.59
PEEP (cmh_2_o)	14 ± 4	14 ± 3	14 ± 5	0.78
Driving pressure (cmh_2_o)	18 ± 7	19 ± 8	18 ± 6	0.84
Fio_2_(%)	100 [92–100]	100 [95–100]	100 [8–100]	0.85
Pao_2_/fio_2_ ratio	67 [59–79]	63 [57–78]	70 [63–81]	0.28
Paco_2_(mmhg)	62 ± 11	61 ± 10	63 ± 12	0.67
pH	7.31 ± 0.10	7.34 ± 0.10	7.28 ± 0.10	0.08
Lactate (mmol/l)	1.7 ± 0.7	1.8 ± 0.9	1.7 ± 0.6	0.65
Norepinephrine use (*n*,%)	10 (42)	3 (30)	7 (50)	0.42
Dose of norepinephrine (μg/kg/min)	0.3 [0.2–0.8]	0.2 [0.1–0.7]	0.3 [0.3–0.8]	0.72
PP sessions (*n*)	4 [3–5]	4 [3–6]	3 [2–4]	0.35
Neuromuscular blockers (*n*,%)	24 (100)	10 (100)	14 (100)	1.00
Inhaled nitric oxide use (*n*,%)	9 (37)	4 (40)	5 (36)	0.99
**Outcomes**				
Duration of VV-ECMO (days)	14 [7–26]	20 [13–31]	9 [4–17]^*^	0.01
Proportion of VV-ECMO weaning (*n*,%)	17 (71)	7 (70)	10 (71)	0.99
Duration of mechanical ventilation (days)	37 [25–51]	41 [31–50]	35 [17–51]	0.27
Length of stay in ICU (days)	41 [28–51]	43 [34–52]	38 [23–50]	0.35
Mortality at Day-28 (*n*,%)	4 (17)	1 (10)	3 (21)	0.61
Mortality at Day-60 (*n*,%)	10 (42)	4 (40)	6 (43)	0.99

*Data are expressed as number (percentages), mean ± standard deviation, or median [interquartile]*.

* *p < 0.05 no PP vs. PP. Driving pressure was calculated as plateau pressure–total positive end-expiratory pressure. FiO_2_, inspired fraction of oxygen; PaO_2_, arterial oxygen partial pressure; PaCO_2_, arterial carbon dioxide partial pressure; PEEP, positive end-expiratory pressure; PP, prone positioning; SAPS, simplified acute physiology score; VV-ECMO, venovenous extracorporeal membrane oxygenation*.

Implantation of VV-ECMO occurred 15 (12–22) days after the onset of symptoms and 6 (3–11) days after the onset of mechanical ventilation. Twenty-three (96%) patients were cannulated using femoro-jugular approach. Before VV-ECMO implantation, all the patients received neuromuscular blocker agents, nine (37%) received inhaled nitric oxide, and PP was performed in all the patients with 4 (3–5) sessions of PP per patient. The duration of VV-ECMO was 14 (7–26) days, while the duration of mechanical ventilation was 37 (25–51) days, and ICU length of stay was 41 (28–51) days. Mortality rates on Day-28 and Day-60 were 17 and 42%, respectively (Table 1).

### Prone Positioning in Patients Under VV-ECMO

Overall, 38 PP sessions were performed in 10 (42%) patients under VV-ECMO, with 3 (2–5) PP sessions per patient. Delay between the first PP session and VV-ECMO implantation was 2 (1–3) days. The mean duration of PP sessions was 17.4 ± 2.1 h. No serious adverse events were reported for all the PP sessions.

Ventilatory settings and patient management before VV-ECMO implantation, as well as delay in VV-ECMO implantation, were not different among patients on whom PP was performed or not (Table 1). The duration of VV-ECMO was significantly longer (20 (13–31) vs. 9 (4–17) days, *p* = 0.01) in patients on whom PP was performed, while the proportion of VV-ECMO weaning was not different in both groups (70 vs. 71%, *p* = 0.99). The duration of mechanical ventilation, ICU length of stay, and Day-28 and Day-60 mortality rates were not different between the two groups of patients (Table 1).

### Effects of Prone Positioning Under VV-ECMO on Respiratory Mechanics and Oxygenation

**Figure 1:**
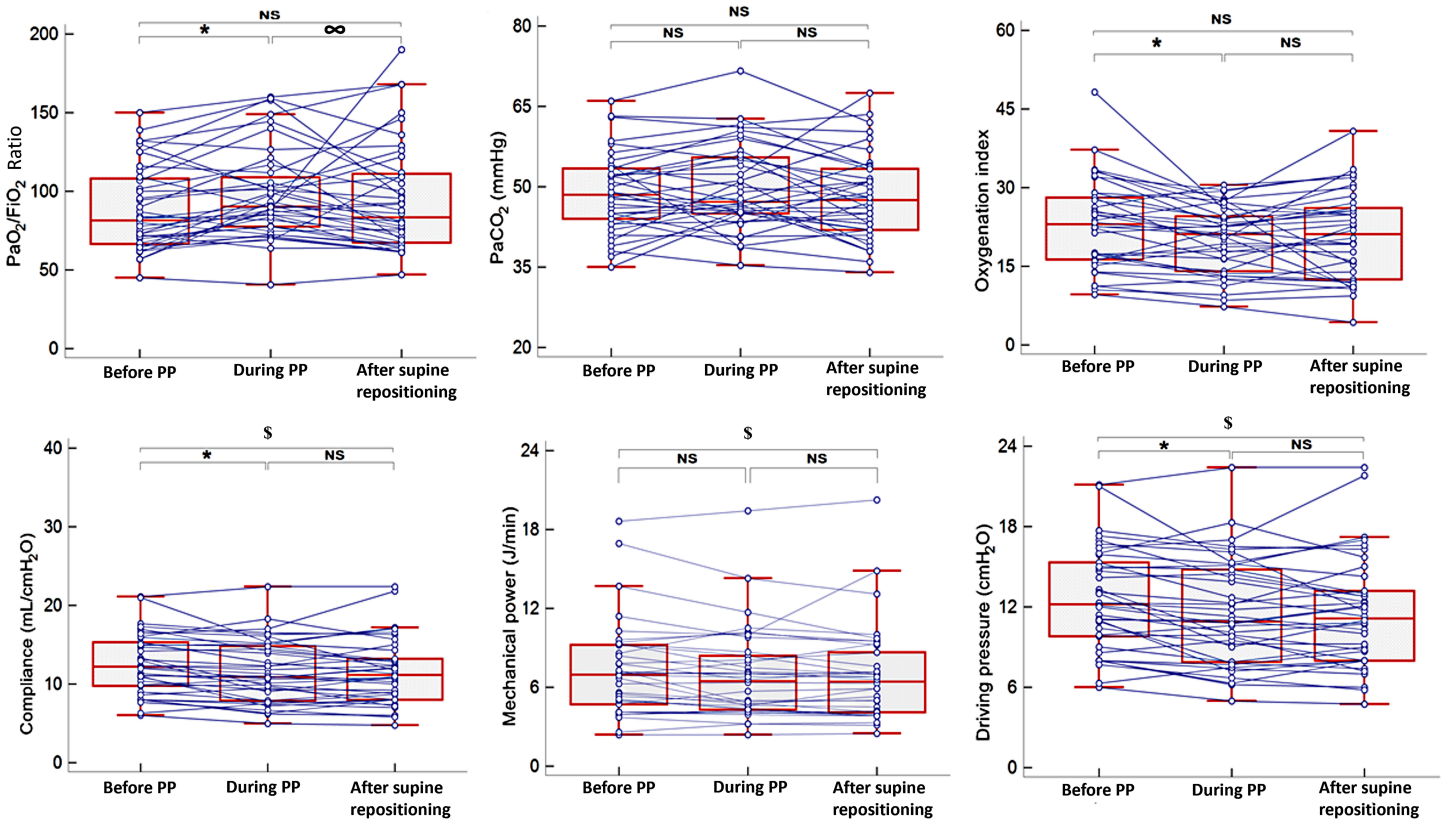
Effects of prone positioning on respiratory mechanical parameters and oxygenation in patients requiring VV-ECMO; n = 38 sessions of prone positioning. The boxes show the 25th and 75th percentiles, the line in the box the median, and the whiskers the minimum and maximum values. The blue lines represent individual changes. *p < 0.05 during vs. before PP; ^$^*p* < 0.05 after supine repositioning vs. before PP; ∞ *p* < 0.05 after supine repositioning vs. during PP. FiO_2_, inspired fraction of oxygen; PaO_2_, arterial oxygen partial pressure; PaCO_2_, arterial carbon dioxide partial pressure; PP, prone positioning; VV-ECMO, venovenous extracorporeal membrane oxygenation.

Under VV-ECMO, PP significantly increased the PaO_2_/FiO_2_ ratio by 14 ± 21% and compliance by 8 ± 15%, and significantly decreased the oxygenation index by 13 ± 18% and driving pressure by 8 ± 12%. The effects of PP on respiratory mechanics but not on oxygenation persisted after supine repositioning (Figure 1, Supplemental Table S1).

**Figure 2:**
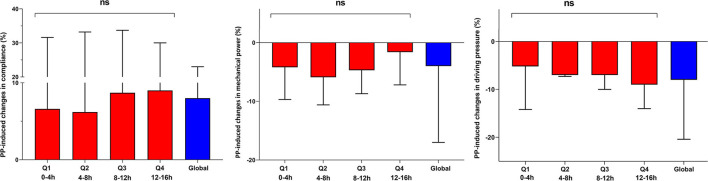
Evolution of respiratory mechanical parameters during prone positioning (PP) sessions in patients requiring veno-venous extra-corporeal membrane oxygenation; *n* = 38 PP sessions. Data are expressed as mean ± standard deviation. The red bars represent PP-induced changes (in percentages from before PP) in respiratory mechanical parameters during the different time quartiles (Q1-Q4) of PP sessions. The blue bars represent global PP-induced changes (in percentages from before PP) in respiratory mechanical parameters. NS, non-significant analysis of variance (ANOVA) *p*-value.

Under VV-ECMO, PP-induced changes in different respiratory mechanical and oxygenation parameters appeared as early as the first-time quartile (increase in PaO_2_/FiO_2_ ratio by 6 ± 22% and decrease in oxygenation index by 15 ± 18%), without any difference in PP-induced changes among the different time quartiles (Figure 2 and Supplemental Table S2).

### Response to Prone Positioning

Among the 38 PP sessions, 13 (34%) induced an increase in PaO_2_/FiO_2_ratio > 20%, and seven (70%) patients were responders to at least one PP session (Supplemental Figure S1). The proportion of PP sessions associated with increase in PaO_2_/FiO_2_ratio > 20% was not different between the PP sessions performed within and after the first 7 days of VV-ECMO implantation (30 vs. 40%, respectively, *p* = 0.73).

**Figure 3:**
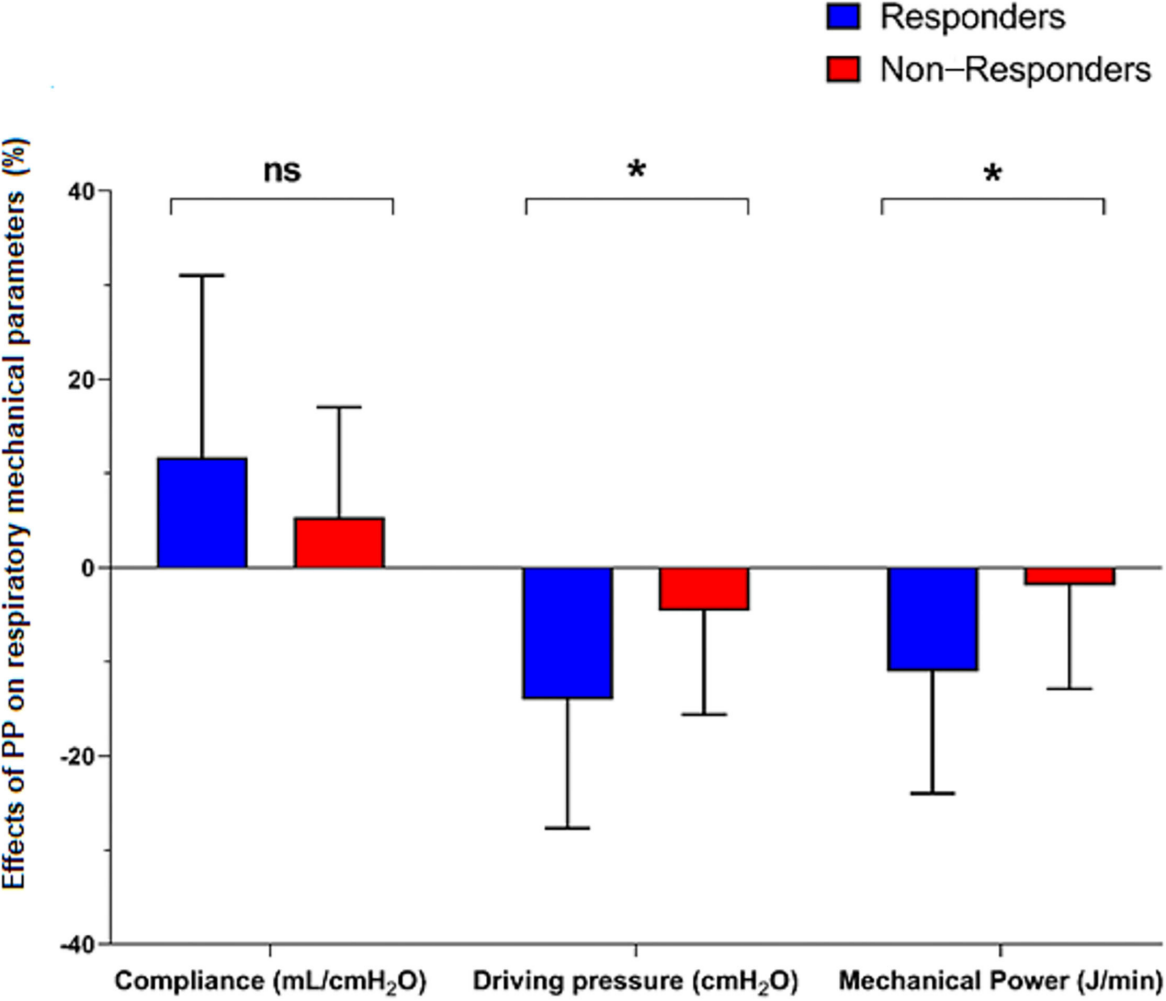
Effects of prone positioning (PP) on respiratory mechanical parameters in responder and non-responder patients, *n* = 38 sessions of PP. Data are expressed as mean ± standard deviation. The bars represent PP-induced changes (in percentages from before PP) in respiratory mechanical parameters in “responders” (increase in PaO_2_/FiO_2_ ratio > 20% during a PP session, blue bars) and “non-responders” (red bars) patients. *p < 0.05 non-responders vs. responders; NS, non-significant. FiO_2_, inspired fraction of oxygen; PaO_2_, arterial oxygen partial pressure.

Prone positioning (PP)-induced changes in driving pressure (−14 ± 14 vs. −6 ± 10%, *p* = 0.04) and mechanical power (−11 ± 13 vs. −0.1 ± 12%, *p* = 0.02) were significantly higher in the responders than in the non-responders, whereas PP-induced changes in compliance were non-different between both groups of patients (12 ± 20 vs. 5 ± 10%, respectively, *p* = 0.28) (Figure 3). PP-induced changes in driving pressure (r = −0.37, *p* = 0.02) and mechanical power (r = −0.38, *p* = 0.02) were significantly correlated with PP-induced changes in PaO_2_/FiO_2_ ratio, whereas PP-induced changes in compliance were not (r = 0.15, *p* = 0.38).

## Discussion

Prone positioning (PP) is a rescue therapy in most severe patients with ARDS, but its effects on respiratory mechanics and prognosis in patients implanted with VV-ECMO have been scarcely investigated so far. In our cohort of patients with COVID-19 and severe ARDS requiring VV-ECMO, PP was performed in 42% of the patients under VV-ECMO without any serious adverse events. Overall, 70% of the patients were responders to at least one PP session (defined by increase in PaO_2_/FiO_2_ratio > 20% during a PP session). Based on the analysis of continuous respiratory data, we showed that PP in patients under VV-ECMO improved oxygenation, and that the respiratory mechanical parameters, as evidenced by increase in compliance of the respiratory system associated with decrease in driving pressure and in mechanical power. The PP effects in patients under VV-ECMO on respiratory mechanical and oxygenation parameters appeared as early as the first-time quartile of a PP session, without any difference in PP-induced changes among the different time quartiles. Unlike the improvement in oxygenation, the effects of PP on the respiratory mechanical parameters persisted after supine repositioning.

To our knowledge, this is the first study investigating the physiological respiratory effects of PP in patients with severe ARDS under VV-ECMO from respiratory parameters that were continuously recorded throughout patient management and PP sessions. Our results confirmed that PP improved the respiratory mechanics of patients under VV-ECMO, as previously shown in severe ARDS related or not to COVID-19 (24–26), by inducing increase in the compliance of the respiratory system and decrease in driving pressure and mechanical power, which are well-known prognostic factors in patients with ARDS (27, 28). We also confirmed that using PP in patients under VV-ECMO improved their oxygenation, as previously shown in patients with severe ARDS related or not to COVID-19 (15, 16, 26, 29). In agreement with existing literature, we also found longer duration of VV-ECMO in patients on whom PP was performed (15, 16), while the proportion of VV-ECMO weaning was not different in both groups (16). A recent meta-analysis found that PP in patients under VV-ECMO improved oxygenation and respiratory mechanics but did not reduce the mortality while increasing ICU length of stay and ECMO duration (30). The potential discrepancy in the PP effects on patients under VV-ECMO with better oxygenation and respiratory mechanics but longer VV-ECMO duration and conflicting results regarding mortality rate and the proportion of VV-ECMO weaning might be partly explained by the fact that PP is preferentially used in more severe patients with persistent hypoxemia under VV-ECMO. The ongoing randomized PRONECMO study (NCT04607551), in which patients with severe ARDS requiring VV-ECMO are randomized for PP sessions, may provide some answers to the effects of PP on mortality rate and outcomes (proportion of VV-ECMO weaning and VV-ECMO duration).

Here, we showed that the effects of PP on respiratory mechanical parameters appeared as early as the first time quartile of a PP session without any difference in PP-induced changes among the different time quartiles. In addition, the PP-induced effects on respiratory parameters persisted after supine repositioning, unlike the improvement in oxygenation. These results suggest that the physiological respiratory effects of PP on patients with severe ARDS requiring VV-ECMO are two-fold. First, PP may improve alveolar recruitment, as evidenced by increase in compliance of the respiratory system and decrease in driving pressure, despite the absence of PaCO_2_ drop. Currently, the effects of PP on alveolar recruitment are still debated both in patients requiring (6, 15, 24, 25, 31, 32) VV-ECMO and those who do not. Second, PP may also improve ventilation/perfusion ratio, as evidenced by loss of PP effect on oxygenation after supine repositioning, despite the persistent effects of PP on alveolar recruitment. Finally, the fact that the patients who were responders to PP in terms of oxygenation had higher decrease in driving pressure than the non-responder patients suggests the potential combined physiological respiratory effects of PP on patients requiring VV-ECMO.

We found that one third of the PP sessions induced an increase in PaO_2_/FiO_2_ ratio > 20%, and that the proportion of PP sessions associated with increase in PaO_2_/FiO_2_ ratio > 20% was not different between the PP sessions performed within and after the first 7 days of VV-ECMO implantation. Our results differ from those in Kimmoun et al., which showed better response to PP when it was performed late (> 7days) after ECMO-VV implantation (24). This discrepancy with our results can be explained as follows. First, the etiology of ARDS was different, with only ARDS related to COVID-19 in our cohort, but pulmonary and non-pulmonary ARDS in the cohort by Kimmoun et al. (24). Second, our patients appeared to be most severe before the PP sessions, with a PaO_2_/FiO_2_ ratio of 83 (69–110) vs. 111 (84–128). Third, the mean duration of PP sessions also differed (17.4 ± 2.1 vs. 24 h).

Finally, we reported no serious adverse events for all the PP sessions. It must be kept in mind that PP sessions in patients under VV-ECMO were performed only by a minimum of five experienced and trained caregivers, and that PP can induce serious adverse effects such as cannula conflicts or bleeding in patients. Only moderate adverse effects were reported in the existing literature, with a proportion ranging from 0 to 21% (24, 29, 33, 34).

We acknowledge some limitations to our study. First, this was a single-center study with a limited number of patients. Nevertheless, we analyzed the respiratory effects of 38 PP sessions from continuously recorded respiratory data, with over a thousand time points during the PP sessions. Second, the decision to perform PP in patients under VV-ECMO was left at the discretion of the attending physician, except in patients with persistent PaO_2_/FiO_2_ ratio < 100, which represented 68% of the PP sessions. All the other PP sessions were performed in patients under VV-ECMO with a PaO_2_/FiO_2_ < 150 and/or with impaired respiratory mechanics. Third, we could not investigate the effects of PP on mortality and outcomes in this physiological and exploratory study. Further randomized studies are needed to confirm our results, and to investigate the effects of PP on the prognosis of patients with severe ARDS requiring VV-ECMO.

## Conclusions

In patients with COVID-19 and ARDS, PP under VV-ECMO was well-tolerated and improved the respiratory mechanical and oxygenation parameters as early as the first-time quartile of a PP session. Unlike the improvement in oxygenation, the effects of PP on the respiratory mechanical parameters persisted after supine repositioning. Further studies are needed to confirm the potential interest of PP in patients with ARDS requiring VV-ECMO.

## Data Availability Statement

The raw data supporting the conclusions of this article will be made available by the authors, without undue reservation.

## Ethics Statement

The studies involving human participants were reviewed and approved by Ethics Committee of the Société de Réanimation de Langue Française (CE SRLF 20-72). The patients/participants provided their written informed consent to participate in this study.

## Author Contributions

DL and MJ designed the study and wrote the original draft. DL, JC, and ZH collected and analyzed data. LN, FP, AC, MJ, and J-PM revised the original draft. All authors contributed to the article and approved the submitted version.

## Conflict of Interest

The authors declare that the research was conducted in the absence of any commercial or financial relationships that could be construed as a potential conflict of interest.

## Publisher's Note

All claims expressed in this article are solely those of the authors and do not necessarily represent those of their affiliated organizations, or those of the publisher, the editors and the reviewers. Any product that may be evaluated in this article, or claim that may be made by its manufacturer, is not guaranteed or endorsed by the publisher.

## Abbreviations

ARDS: acute respiratory distress syndrome
FiO_2_: inspired fraction of oxygen
PaCO_2_: arterial carbon dioxide partial pressure
PaO_2_: arterial oxygen partial pressure
PEEP: positive end-expiratory pressure
P_plat_: plateau pressure
PP: prone positioning
VV-ECMO: venovenous extracorporeal membrane oxygenation.
