# Supplementary figures and images for: Effects of Prone Positioning on Respiratory Mechanics and Oxygenation in Critically Ill Patients With COVID-19 Requiring Venovenous Extracorporeal Membrane Oxygenation
Source: Front Med (Lausanne). 2022 Jan 17;8:810393. doi: 10.3389/fmed.2021.810393 (PMC8801420; doi:10.3389/fmed.2021.810393)

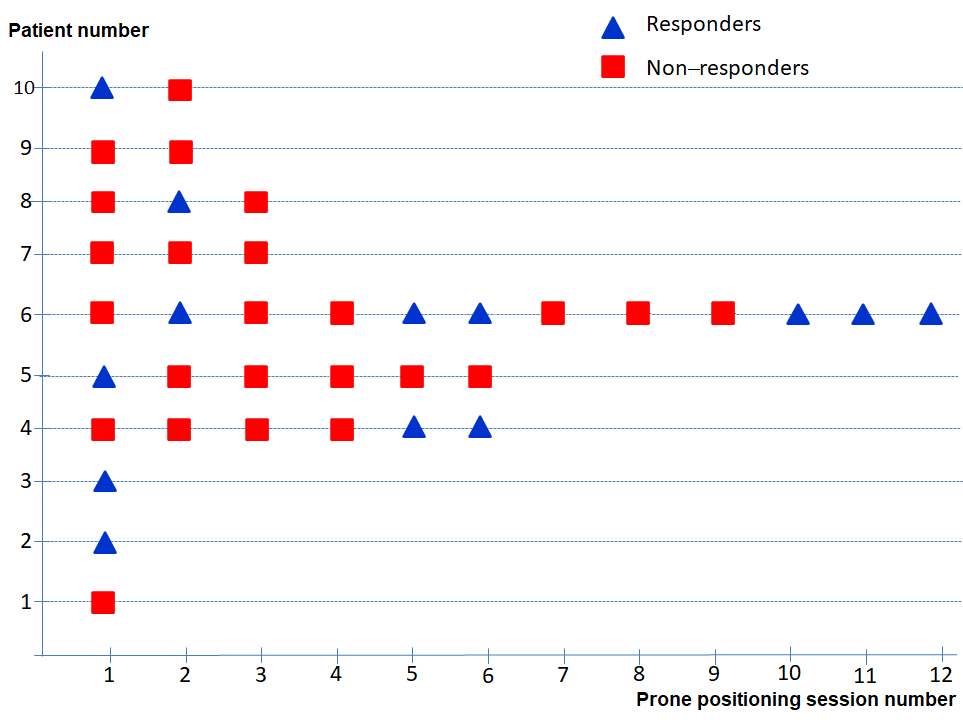

Supplement: Supplementary file 1 [file Image_1.TIF]
