# Supplementary material for: Effects of Prone Positioning on Respiratory Mechanics and Oxygenation in Critically Ill Patients With COVID-19 Requiring Venovenous Extracorporeal Membrane Oxygenation
Source: Front Med (Lausanne). 2022 Jan 17;8:810393. doi: 10.3389/fmed.2021.810393 (PMC8801420; doi:10.3389/fmed.2021.810393)
Supplement: Supplementary file 2 [file Table_1.DOCX]

**Effects of prone positioning on respiratory mechanics and oxygenation in critically-ill patients with COVID-19 requiring venovenous extracorporeal membrane oxygenation**

Driss LAGHLAM MD^1,2^, Julien CHARPENTIER MD^1,2^, Zakaria AIT HAMOU MD^1,2^, Lee NGUYEN MD, PhD^3^, Frédéric PENE MD, PhD^1,2^, Alain CARIOU MD, PhD^1,2^, Jean-Paul MIRA MD, PhD^1,2^ Mathieu JOZWIAK MD, PhD^1,2,4^

1: Assistance Publique – Hôpitaux de Paris, Hôpitaux universitaires Paris-Centre, Hôpital Cochin, Service de Médecine Intensive Réanimation, 27, rue du faubourg Saint Jacques, Paris, F-75014, France

2: Université de Paris, Paris, France

3: RICAP- Clinique Ambroise Paré, 25-27 boulevard Victor Hugo Neuilly-Sur-Seine

4 : Equipe 2 CARRES, UR2CA - Unité de Recherche Clinique Côte d'Azur, Université Côte d’Azur, Nice, France

**Corresponding author:**

Driss LAGHLAM, MD

Assistance Publique – Hôpitaux de Paris, Hôpitaux universitaires Paris-Centre

Service de Médecine Intensive Réanimation, hôpital Cochin

27 rue du Faubourg Saint Jacques

75014 Paris, France

Mail: [driss.laghlam@aphp.fr](mailto:driss.laghlam@aphp.fr)

Phone number: +33158414145

Fax number: +33158412505

**Supplementary Figure legends**

**Figure S1 - Response to prone positioning sessions for each patient.**

n=38 sessions of prone positioning. Patients with an increase in PaO_2_/FiO_2_ ratio >20% during the prone positioning session were considered as responders. Abbreviations: FiO_2_: inspired fraction of oxygen; PaO_2_: oxygen arterial partial pressure.

**Supplementary Tables**

**Table S1. Effects of prone positioning on respiratory mechanical parameters, oxygenation and hemodynamics in patients requiring VV-ECMO.**

|  | Before PP | During PP | After supine repositioning | *ANOVAp-value* | Percent changes During *vs.* Before PP | Percent changes  After supine repositioning *vs.* Before PP | Percent changes  After supine repositioning *vs.* During PP |
| --- | --- | --- | --- | --- | --- | --- | --- |
| PaO_2_/FiO_2_ ratio | 83 [69-110] | 91 [81-113]* | 87 [69-115] ∞ | 0.02 | +14±21 | +7±37 | -3±30 |
| PaCO_2_ (mmHg) | 49±7 | 50±8 | 48±8 | 0.24 | +2±11 | -2±13 | -3±12 |
| Oxygenation index | 23±8 | 20±6* | 21±8 | 0.03 | -13±18 | -11±30 | +4±27 |
| Compliance (mL/cmH_2_O) | 15±6 | 16±7* | 17±7^$^ | <0.0001 | +8±15 | +11±14 | +4±15 |
| Mechanical power (J/Min) | 6.9 [4.6-9.2] | 6.5 [4.3-8.5] | 6.4 [4.1-8.8] ^$^ | 0.03 | -5±13 | -6±19 | -1±12 |
| Driving pressure (cmH_2_O) | 12.5±4 | 11.6±4* | 11.5±4^$^ | 0.03 | -8±12 | -9±16 | -1±13 |
| Lactate (mmol/L) | 1.1 [0.8-1.5] | 1.1 [0.7-1.4] | 1.0 [0.7-1.4] | 0.21 | -7[-16-7] | -17[-21-16] | -8[-14-22] |
| Heart rate | 111±15 | 109±17 | 106±18 | 0.72 | -1±12 | -4±11 | -3±10 |
| MAP | 81±11 | 79±8 | 78±11 | 0.06 | -1±16 | -3±23 | -2±5 |

n=38 sessions of prone positioning. Data are expressed as mean ± standard deviation or median [interquartile].
*p<0.05 during *vs.* before PP, ^$^p<0.05 after supine repositioning *vs.* before PP, ∞ p<0.05 after supine repositioning *vs.* during PP.

The compliance of the respiratory system was calculated as tidal volume/(plateau pressure – total positive end-expiratory pressure). The driving pressure was calculated as plateau pressure – total positive end-expiratory pressure. The mechanical power was calculated as 0.098 x tidal volume x respiratory rate x peak pressure – driving pressure/2. The oxygenation index was calculated as (mean airway pressure x FiO2)/PaO2.

Abbreviations: FiO_2_: inspired fraction of oxygen; MAP: mean arterial pressure; PaO_2_: arterial oxygen partial pressure; PaCO_2_: arterial carbon dioxide partial pressure, PP: prone positioning, VV-ECMO: venovenous extracorporeal membrane oxygenation

**Table S2.  Evolution of respiratory mechanical parameters and oxygenation during the different time quartiles of prone positioning sessions in patients requiring VV-ECMO.**

|  | Quartile 1  0-4h | Quartile 2  4-8h | | Quartile 3  8-12h | Quartile 4  12-16h | *ANOVA*  *p-value* |
| --- | --- | --- | --- | --- | --- | --- |
| PaO_2_/FiO_2_ ratio | 88 [76-114] | 93 [78-104] | | 90 [79-106] | 94 [78-119] | 0.31 |
| PaCO_2_ (mmHg) | 48±10 | | 51±9 | 52±8 | 49±7 | 0.09 |
| Compliance (mL/cmH_2_O) | 16±8 | 16±7 | | 17±8 | 17±7 | 0.21 |
| Driving pressure (cmH_2_O) | 12±4 | 12±4 | | 12±4 | 11±4 | 0.27 |
| Oxygenation index | 21±8 | 19±8 | | 21±6 | 19±7 | 0.16 |
| Mechanical power (J/Min) | 6.7 [4.4-8.4] | 6.9 [4.1-7.8] | | 6.9 [4.3-8.6] | 6.7 [4.1-9.5] | 0.47 |

n=38 prone positioning sessions. Data are expressed as mean ± standard deviation or median [interquartile]. The compliance of the respiratory system was calculated as tidal volume/(plateau pressure – total positive end-expiratory pressure). The driving pressure was calculated as plateau pressure – total positive end-expiratory pressure. The mechanical power was calculated as 0.098 x tidal volume x respiratory rate x peak pressure – driving pressure/2. The oxygenation index was calculated as (mean airway pressure x FiO2)/PaO2. Abbreviations: FiO_2_: inspired fraction of oxygen; PaO2: arterial oxygen partial pressure; PaCO_2_: arterial carbon dioxide partial pressure; VV-ECMO: venovenous extracorporeal membrane oxygenation.
